# Development of a core collection for ramie by heuristic search based on SSR markers

**DOI:** 10.1080/13102818.2014.953768

**Published:** 2014-11-03

**Authors:** Ming-Bao Luan, Zi-Zheng Zou, Juan-Juan Zhu, Xiao-Fei Wang, Ying Xu, Qing-Hua Ma, Zhi-Min Sun, Jian-Hua Chen

**Affiliations:** ^a^ Chinese Academy of Agricultural Sciences, Institute of Bast Fiber Crops, Key Laboratory of Stem-fiber Biomass and Engineering Microbiology (Ministry of Agriculture), Changsha, Hunan, P. R. China; ^b^ Department of Basic Medicine, Yiyang Medical School,Yiyang, Hunan, P. R. China; ^c^ Department of Plant Nutrition, China Agricultural University,Beijing, P. R. China

**Keywords:** ramie, core collection, SSR

## Abstract

There are more than 2000 ramie germplasms in the National Ramie Germplasm Nursery affiliated with the Institute of Bast Fiber Crops, Chinese Academy of Agricultural Science, China. As it is difficult to perform effective conservation, management, evaluation, and utilization of redundant genetic resources, it is necessary to construct a core collection by using molecular markers. In this study, a core collection of ramie consisting of 22 germplasms was constructed from 108 accessions by heuristic search based on 21 Simple Sequence Repeat (SSR) marker combinations. The results showed that there is a poor relationship between the core collection and the geographic distribution. The number of amplification bands for the core collection was the same as that for the entire collection. Shannon's index for three of the SSR primers (14%) and Nei's index for nine of the SSR primers (19%) were lower in the core collection than in the entire collection. The true core collection had wider genetic diversity compared with the random core collection. Collectively, the core collection constructed in this study is reliable and represents the genetic diversity of all the 108 accessions.

## Introduction

Germplasm collections exist to conserve the genetic diversity of crop species and their wild relatives. Redundant genetic resources result in difficulties in the effective conservation, management, evaluation, and utilization of germplasms. To solve these challenges caused by the large amount of germplasm collections, a limited number of genetically diverse accessions within a collection are usually selected as a core collection, i.e. a representative subset of the entire germplasm collection, consisting of introduced accessions with minimum genetic redundancy and retaining most of the initial collection [1] and given priority in evaluation and hybridization.[2] The core collection provides preliminary information on the diversity available in the larger collection.[3] At present, core collections have been developed not only in annual plants like rice,[4] maize,[5] cotton [6] and wheat,[7] but also in perennial plants such as *Prunus mume* Sieb.,[8] robusta coffee,[9] peach,[10] Chinese tea,[11] *Betula platyphylla* [12] and persimmon.[13]

Ramie (*Boehmeria nivea* L.), also called Chinese grass, is a popular perennial plant native to eastern Asia, a centre of origin for ramie, having the largest amount of genetic resources in the world. Ramie is thought to have been used for at least six thousand years, which makes it one of the oldest fibre crops. It is used principally for fabric production and the part used is the bark (phloem) of the vegetative stalks, from which bast fibre is obtained. A series of ramie products such as shirts, underwear, and health-care socks has been developed and is sold on the home markets. In recent years, ramie is used to conserve soil and water in Yangtze regions, in the south of China [14]; and is also widely cultivated as a forage crop in China.[15,16] Nowadays, more than 2000 accessions of ramie cultivars are preserved in the National Ramie Germplasm Nursery affiliated with the Institute of Bast Fiber Crops, Chinese Academy of Agricultural Science (CAAS), China. Due to the large number of cultivars and redundant genetic resources, it is difficult to determine precisely and rapidly useful resources for plant breeders. It is, therefore, important to construct a core collection of ramie.

Luan et al. [17] published the first data on constructing a core collection of ramie based on agronomic traits. This involved a large project for collecting data of all resources over a multitude of years and sites. To characterize and effectively evaluate ramie in China, molecular markers such as Random Amplified Polymorphic DNA (RAPD),[18–20] Simple Sequence Repeats (SSR),[21–24] Inter-Simple Sequence Repeats (ISSR),[25–27] Sequence-Related Amplified Polymorphism (SRAP),[24,28,29] Restriction Site Amplification Polymorphism (RSAP) [24] and Random Amplified Microsatellite Polymorphism (RAMP) [23] have been employed. Among these markers, SSRs are highly polymorphic, informative, codominant, technically simple, and reproducible, and have become common in constructing core collections.[30] In 2011, our group [31] constructed a primary core collection consisting of 158 germplasms, based on data for 25 agronomic traits from 790 ramie germplasms of the National Ramie Germplasm Nursery. The size of the primary core collection is still quite large and redundancy of some accessions may occur because ramie is clonally propagated. This leads to limitations making its use still time-consuming and inconvenient due to low polymorphism on agronomic traits in used the field. Therefore, it is necessary to develop a core collection with the same genetic diversity as the whole collection, but smaller in size.

There are several advantages in constructing a core collection based on SSR markers as follows. First, there is high polymorphism in SSR markers, which produce a number of amplification bands. Second, the SSR test is simple and without seasonal restrictions. Finally, the statistical analysis of the SSR amplification bands is relatively easy and shows high repeatability, i.e. the method is reliable. All this makes it much less time- and labour-consuming for researchers to construct core collections using the SSR marker approach.

The objective of this study was to develop a core collection from a primary core collection by SSR markers, providing the basis for constructing a core collection by further using of a large number of accessions.

## Materials and methods

### Materials and DNA isolation

A total of 108 ramie accessions (Table 1) grown in the National Ramie Germplasm Nursery affiliated with the Institute of Bast Fiber Crops, CAAS, China were used in this study. DNA was isolated from young leaves collected from each ramie accession. The DNeasy plant mini prep kit (Qiagen, Germany) was used for DNA isolation.

**Table 1.** List of 108 germplasms.

Code	Entry	Origin	Code	Entry	Origin
1	Wayaozhuma	Guangxi	55	Xinminqingma	Guizhou
2	Ganzaerhao	Jiangxi	56	Youqima	Hunan
3	Miaobazhuma	Sichuan	57	Baiyema	Jiangxi
4	Simaohongzhuma	Yunnan	58	Lichuanhoupizhuma	Jiangxi
5	Shuiqingzhuma	Chongqing	59	Boyanghuangyema	Jiangxi
6	Yichuntongpiqing	Jiangxi	60	Xiaoqinggan	Sichuan
7	Yachibaima	Guizhou	61	Yihuangjiama	Jiangxi
8	Rongchangzhuma	Chongqing	62	Xiangningdayelv	Hubei
9	Guangdonghuangpidouerhao	Guangdong	63	Yedouzi	Jiangxi
10	Qingyezhuma	Unkown	64	Tiantaitiema	Zhejiang
11	Hexianjiama	Guangxi	65	Dayehongzhameng	Jiangxi
12	Guangpima	Jiangxi	66	Jiangkouqingpizhuma	Guizhou
13	Juandongtuma	Chongqing	67	Longtangbaima	Chongqing
14	Limuqingma	Guizhou	68	Wuchangshanpozhumayihao	Hubei
15	Dadaoma	Guizhou	69	Jinpingqingma	Guizhou
16	Lipingqingma	Guizhou	70	Qingpigan	Jiangxi
17	Lidazhuma	Yunnan	71	Fenyihuangguangdou	Jiangxi
18	Gebuqingma	Guangxi	72	Huangjiuma	Hunan
19	Tongmuqingma	Guangxi	73	Guangpima	Jiangxi
20	Qingpidamayihao	Chongqing	74	Qianzhuyihao	Guizhou
21	Gaoanma	Jiangxi	75	Loushanhuangpima	Guizhou
22	Yujiangma	Jiangxi	76	Anlongzhumaerhao	Sichuan
23	Fulisima	Chongqing	77	Linggongqingpima	Guizhou
24	Wuchuanbaima	Guizhou	78	Japanzhumaqihao	Japan
25	Huanhancongma	Sichuan	79	Xieliqingma	Chongqing
26	Nanchongzhuma	Sichuan	80	Kuguaqing	Hunan
27	Xiningxianma	Chongqing	81	Longhuibaimayihao	Hunan
28	Puqidayelvyihao	Hubei	82	Yongshanzhuma	Yunnan
29	Wulonghonggan	Chongqing	83	Honggujin	Hubei
30	Xiaogubai	Jiangxi	84	Yushanma	Jiangxi
31	Rongjiangbaimayihao	Guizhou	85	Yinniyihao	Indonesian
32	Niutima	Hunan	86	Sichuangaodibaima	Chongqing
33	Nanchenghoupizhuma	Jiangxi	87	Yinnierhao	Indonesian
34	Huangqingdou	Jiangxi	88	Shanqingbaima	Chongqing
35	Zixima	Jiangxi	89	Ershiqingmaerhao	Hubei
36	Datianhuangganzhuma	Yunnan	90	Xinningqingma	Hunan
37	Ningduyema	Jiangxi	91	Ximatuma	Guangxi
38	Heipima	Guangxi	92	Yinnisanhao	Indonesian
39	Tianbaoma	Jiangxi	93	Zunyichuangenma	Guizhou
40	Changshaqingyema	Hunan	94	Dayujiandaobai	Hubei
41	Chuanzhuerhao	Sichuan	95	Xiaoyelugan	Jiangxi
42	Nanchengbaopizhuma	Jiangxi	96	Pingchangjiama	Sichuan
43	Ningduqingzhuma	Jiangxi	97	Leiyanghuangkema	Hunan
44	Manqiangzhuma	Hubei	98	Gaodiqingma	Chongqing
45	Lvzhubai	Jiangxi	99	Dingyezhuma	Guangxi
46	Dayujiandaobai	Hubei	100	Kanlazhuma	Myanmar
47	Quxianzhuma	Zhejiang	101	Shengbaxianma	Hubei
48	Zhuzibian	Jiangxi	102	Xinpuqingma	Guizhou
49	Yangshuojigubai	Guangxi	103	Tianpaishanyema	Guangxi
50	Hupima	Jiangxi	104	Huangjindou	Hubei
51	Yangxinxiyelv	Hubei	105	Chongrenzhuma	Hunan
52	Xiangzhuliuhao	Hunan	106	Gegenma	Hunan
53	Ningdudabaima	Jiangxi	107	Tianbaoma	Jiangxi
54	Xiangzhuyihao	Hunan	108	Huangpinghuangganma	Guizhou

### SSR primers

Twenty-one SSR primer pairs (Table 2) were synthesized according to Chen et al.[32]

**Table 2.** Sequences of SSR primers used in the genetic assessment of ramie accessions.

Locus	Primer sequences (5^′^–3^′^)	Primer sequences (5^′^–3^′^)
b50	F: AACAATCCAGGAGTGGCAATC	R: ACAAGCGAAGATCGTCTCATC
b35	F: CGTTCAGTCACCAGCAAGG	R: GAGGGAAGCAGGGAGAGC
b38	F: TAATCCCTCAATGGCTCTTTTC	R: GAGAAGGATACGAATTGACAGG
b40	F:TGTATAGAACTGAGTAAATGATTG	R: CAACTTTCTTAAACCACTTTCG
b43	F: CGAGCCTTCTTCTTCTTCTGG	R: GCAAGCAATACGGACAGTAGG
c03	F:CGTGAAAATAGTGATATGTGTG	R: ACTGTAACAATCAAGAAGAAACC
c07	F:GCCACAGCCGAGGAAGAG	R: TCTCATCACCACCACCTTAGG
b27	F: AGCCAGGTTCCAGAAGTCC	R: CATAATCACAAAGTCTCGGTTCC
b28	F: TCCCACCACGGACTACTG	R: AACCACCATCATCATCATCATC
b11	F: GCGGAGGCTTAATTTGCTTTG	R: ACTCAATACATACACGGCACTAG
b16	F: ACCTCTACGGACCTCTTCTTC	R: CATAACATAACATGACACACAAGC
b24	F: GAGCCAGAGCCAGGTTCC	R: ACAAAGTCTCGGTTCCTTACAC
b34	AATAGAATGTGGAGGCGATAGAG	R: AAACCATAAATCAACTACCGAACC
b64	F: CTTGAGATACAGCCTTCCATTAG	R: CACACCTCGCTTCCCTTG
c17	F:GAAACTATTTCCACCAACAAAG	R: ACACACATTCCTACACACC
b57	F: CGGATATGGTGGAGGTTATGC	R: CAGAACGACGACGACGAC
b65	F: ACGAACCACAACACAGAGAG	R: ACGAGGGAACACCAGAGAG
c18	F:AAGCCGAGCGTGAAGAAG	R: ACACACAGAAAGAACACAAGAC
b53	F: GGCTCAAGTTTGCTCATAGATTC	R: CGGCTTCGCTTTAGGATTTG
b56	F: CGGTCTGTGGATACGAATGG	R: GACGACGACGACGATGATG
c10	F:AGTGCGGAGATAACTGTTC	R: GGCTACTTTATTCTAAACCAAAC

### SSR analysis

SSR-primed polymerase chain reactions (PCRs) were carried out in 10 μL reaction volumes with 1×PCR buffer, 0.2 mmol/L dNTP, 1 U Taq DNA polymerase (Tiangen), 0.5 μL of forward primer (10 nmol/L), 0.5 μL of reverse primer (10 nmol/L), and 0.5 μL of DNA from each accession. PCR was performed under the following conditions, 94 °C for 5 min, followed by 30 cycles of 30 s at 95 °C, 30 s at the primer-specific annealing temperature, 30 s at 72 °C, and a final extension of 10 min at 72 °C.

The PCR products were separated in 8% polyacrylamide gels, and silver dyeing was conducted according to Zhang et al.[33] Molecular weights were estimated using a DNA marker (DNA Marker 2000, BioTeke Co., Beijing, China). Clear bands were recorded as 1 and the absence of bands as 0, and the electrophoresis results were represented as a binary matrix. Amplification band types were recorded. SSR analysis was repeated at least twice.

### Data analysis

Observed heterozygosity (H_O_) and expected heterozygosity (H_E_) were determined by using Popgen 1.31 software.[34]

Powercore software was used to construct the core collection.[35] It can select candidate entries by calculating the costs to reach the goal. Therefore, even if the selection of subsets is repeated using the same data, only one core size is generated.

The genetic similarity matrix was obtained using the SIMQUAL sub-routine of the NTSYS-pc software statistical package [36] based on Jaccard's algorithms.

### Evaluation criteria for the core collection

The representativeness of the core collection was validated according to the following criteria. The coincidence rate of the band types of SSR markers was defined as the percentage of bands of SSR markers amplified in the core collection versus that in the primary core collection; the coincidence rate of Shannon's index was calculated as the ratio between Shannon's index in the core collection and that in the primary core collection; the coincidence rate of Nei's index was calculated as the ratio between Nei's index in the core collection and that in the primary core collection. The appropriate core collection should bear at least 70% of the genetic diversity of the entire collection.

## Results and discussion

Ramie germplasm resources are of great importance in breeding, scientific research, teaching, and production. Traditionally, its germplasm resources are conserved in the field, but there is always damage due to the spread of diseases among germplasm resources and to natural disasters. For instance, the extraordinarily serious flood of 1996, which overflowed the nursery of the national ramie germplasm collection, resulted in the spread of bacterial wilt, which caused enormous losses of ramie germplasm resources.[37] It is necessary, therefore, to back-up the germplasm collection to reduce the risks of damage. However, a major difficulty is that ramie is a clonal crop and the germplasms cannot be conserved via seed propagation. The technology of conservation is more complex in clonal crops than in seed crops and the redundancy is larger in clonal crops than in seed crops.[30] All this makes the cost of management very high, causing severe restrictions. A core collection, being a subset of a large germplasm collection, allows the maximum possible genetic diversity of a crop species to be preserved with minimum redundancy.

Some species have core collections constructed using SSR markers. For example, Zhang et al. [30] constructed the core collection of Japanese persimmon by using SSR markers. At present, to the best of our knowledge, there is no report related to core collection construction of ramie, using SSR markers. Thus, in this study, a core collection of ramie was constructed based on SSR markers in order to back-up the entire germplasm collection of ramie.

### Genetic diversity in the primary collection

The first step in our study was to analyse the genetic diversity in the primary collection. Table 3 shows that the number of alleles per locus ranged from two to three at the tested 21 loci. Fourteen primer pairs amplified two alleles, while the remaining seven primer pairs amplified three alleles. The observed heterozygosity (H_O_) over all tested loci ranged from 0.23 to 0.93 and the expected heterozygosity (H_E_) ranged from 0.21 to 0.64.

**Table 3.** Number of alleles, observed and expected heterozygocity across 21 SSR loci.

Locus	Number of alleles	Observed heterozygocity	Expected heterozygocity
b50	2	0.23	0.40
b35	3	0.72	0.53
b38	2	0.48	0.39
b40	3	0.37	0.45
b43	2	0.40	0.32
c03	2	0.46	0.50
c07	2	0.93	0.50
b27	2	0.35	0.45
b28	2	0.29	0.41
b11	2	0.34	0.50
b16	2	0.46	0.46
b24	2	0.34	0.47
b34	2	0.28	0.40
b64	2	0.24	0.21
c17	2	0.58	0.41
b57	2	0.59	0.50
b65	3	0.37	0.42
c18	3	0.67	0.64
b53	3	0.65	0.60
b56	3	0.59	0.61
c10	3	0.51	0.64

### Construction of the core collection

In this study, the core collection of ramie was constructed by using of heuristic search based on SSR markers. Heuristic search is a method of state-space search that estimates each search node until finding the best one, and then searches sequentially from this best node until finding the goal. That is, state-space search is a problem-solving process that aims to find the optimal path from an initial state to the goal state. Thus, the core collection constructed using heuristic search is unique.

The core collection included 22 ramie germplasms (Table 4), of which 7 germplasms were collected from Guizhou Province, 6 germplasms from Jiangxi Province, 4 germplasms from the municipality of Chongqing city, 2 germplasms from Guangxi Province, and the rest of the ramie germplasms from Yunnan, Sichuan, and Hubei Province, respectively. Their geographic distribution indicated that there was poor relationship between the core collection and the geographic distribution.

**Table 4.** List of core collection accessions.

Germplasm	Origin
Ganzaerhao	Jiangxi
Shuiqingqingma	Chongqing
Hexianjiama	Guangxi
Dadaoma	Guizhou
Lipingqingma	Guizhou
Fulisima	Chongqing
Wuchuanbaima	Guizhou
Datianhuangganzhuma	Yunnan
Tianbaoma	Jiangxi
Chuanzhuerhao	Sichuan
Lvzhubai	Jiangxi
Zhuzibian	Jiangxi
Xinmingqingma	Guizhou
Lichuanhoupizhuma	Jiangxi
Jiangkouqingpizhuma	Guizhou
Qianzhuyihao	Guizhou
Sichuangaodibaima	Chongqing
Shanqingbaima	Chongqing
Ximatuma	Guangxi
Shengbaxianma	Hubei
Tianbaoma	Jiangxi
Huangpihuangganma	Guizhou

### Evaluation of the core collection

Table 5 shows the number of amplification bands produced by the 21 SSR primer pairs in the entire collection and the core collection, respectively. The number of amplification bands in the core collection was the same as that in the entire collection, suggesting that all belt types of the entire accession were contained in the core collection. Shanon's index and Nei's index of the core collection and the entire collection were calculated, respectively. Of 21 SSR primer pairs, the Shanon Index for three SSR primers was lower in the core collection than in the entire collection, accounting for 14% of all the SSR primers used. Nei's index for four SSR primers was also lower in the core collection than in the entire collection, accounting for 19% of all the SSR primers used. When the core collection retains at least 70% of the genetic diversity of the entire collection, it could be considered satisfactory. Thus, the core collection developed in this study could be considered to reliably represent the genetic diversity of the entire collection.

**Table 5.** Genetic diversity in core collection and entire collection.

	Core collection			Entire collection			Core/entire		
Locus	Shannon	Nei	BT^a^	Shannon	Nei	BT	Shannon	Nei	BT
b50	1.151	0.64	9	1.078	0.582	9	1.07	1.10	1.00
b35	1.491	0.756	5	1.41	0.73	5	1.06	1.04	1.00
b38	1.123	0.628	4	0.908	0.552	4	1.24	1.14	1.00
b40	1.728	0.793	7	1.35	0.67	7	1.28	1.18	1.00
b43	0.837	0.533	3	0.716	0.486	3	1.17	1.10	1.00
c03	1.448	0.731	5	1.378	0.714	5	1.05	1.02	1.00
c07	0.663	0.318	4	0.422	0.188	4	1.57	1.69	1.00
b27	1.024	0.566	4	1.087	0.628	4	0.94	0.90	1.00
b28	1.503	0.736	6	1.336	0.682	6	1.13	1.08	1.00
b11	1.227	0.69	4	1.167	0.676	4	1.05	1.02	1.00
b16	1.207	0.678	4	1.145	0.642	4	1.05	1.06	1.00
b24	1.123	0.628	4	1.102	0.648	4	1.02	0.97	1.00
b34	1.184	0.616	5	1.167	0.615	5	1.01	1.00	1.00
b64	0.586	0.397	2	0.541	0.356	2	1.08	1.12	1.00
c17	0.625	0.434	2	0.679	0.486	2	0.92	0.89	1.00
b57	1.19	0.665	4	1.25	0.672	4	0.95	0.99	1.00
b65	1.54	0.719	7	1.243	0.624	7	1.24	1.15	1.00
c18	1.934	0.839	8	1.778	0.776	8	1.09	1.08	1.00
b53	1.772	0.81	7	1.568	0.728	7	1.13	1.11	1.00
b56	1.918	0.835	8	1.795	0.811	8	1.07	1.03	1.00
c10	1.811	0.818	7	1.815	0.821	7	1.00	1.00	1.00

^a^BT: band type.

Additionally, a random core collection consisting of 22 ramie germplasms was selected and the calculations were repeated three times. The similarity coefficients between the random core collection and the true core collection in this study were compared. The results are shown in Table 6. The similarity coefficient between the entire collection and the random core collection or the true core collection ranged from 0.48 to 0.87 and from 0.32 to 0.78, respectively, suggesting that the true core collection had wider genetic diversity compared with the random core collection. These results further confirmed that the core collection constructed in this study was reliable.

**Table 6.** Similarity coefficients of the true and random core collection.

Collection	Max	Min
True core collection	0.32	0.78
Random core collection	0.48	0.87

### Final remarks

Ramie, being a cash crop in China, plays an important role in natural fibre production. In recent years, with the expansion of its field of applications, ramie is used in medicine,[38] feedstuffs,[15,16] soil and water conservation,[14] industrial raw materials, and bio-fuels.[15] For example, there are significant achievements on the medicinal value of its leaves and roots.[38] Its leaves can also be used as animal feed and the trials have entered the stage of pilot scale.[15] Moreover, the Chinese Ministry of Water Resources has demonstrated that ramie is one of the most effective crops for conserving soil and water in the south hilly region.[14] As the germplasm resource basis for scientific research, it is essential to expand the knowledge on the genetic effects and to locate genes of multiple-use ramie. However, it is rather complicated and time-consuming to screen for genes associated with a specific use of ramie because of the very large number of germplasms. A core collection representing the entire genetic diversity of germplasm resources can improve the identification efficiency. For example, Holbrook and Anderson [39] identified leaf-spot resistance in peanut, using a core collection, which took twice as less time than screening the whole germplasm. Jiang et al. [40] also used a core collection as a less time-consuming approach to identify peanut germplasm with resistance to *Aspergillus flavus*. Therefore, building a ramie core collection can lay a solid foundation for exploring new applications of ramie.

In this study, only 108 accessions were used because SSR molecular markers were first employed in constructing a core collection of ramie. The next step will be to widen the amount of ramie germplasms. The results of this study provide the basis for further improvement of the core collection.

## Conclusions

To the best of our knowledge, this study is the first to use SSR molecular markers to construct a core collection of ramie. The core collection constructed on the basis of SSR markers showed the same number of amplification bands as the entire collection, suggesting that it represents the genetic diversity of the entire collection. The constructed core collection is of great significance for ramie germplasm conservation, evaluation, and utilization. The next step will be to widen the amount of ramie germplasms. The results of this study provide the basis for further improvement of the core collection.
